# Real-world effects of incretin-based Obesity medications on body composition

**DOI:** 10.1016/j.obpill.2025.100229

**Published:** 2025-11-10

**Authors:** Rahila Bhatti, Amena Sadiya, Bashair M. Mussa, Rawoof Khan, Salah Abusnana

**Affiliations:** a Endocrinology Department, Genesis Healthcare Centre, Dubai, United Arab Emirates; b Clinical Science Department, College of Medicine, University of Sharjah, Sharjah, United Arab Emirates; c Basic Medical Science Department, College of Medicine, University of Sharjah, Sharjah, United Arab Emirates; d Diabetes and Endocrinology Department, University Hospital Sharjah, Sharjah, United Arab Emirates

**Keywords:** Incretin-based medications, Semaglutide, Tirzepatide, Obesity management, Body composition, Weight loss, Metabolic parameters, Real-world study

## Abstract

**Background:**

This study evaluated the real-world impact of incretin-based obesity medications Semaglutide and Tirzepatide on body composition in people with obesity. The primary outcomes included changes in weight, waist circumference, skeletal muscle mass, fat mass, and visceral fat over 12 months.

**Methods:**

A retrospective observational study was conducted at Genesis Healthcare Centre, Dubai, UAE, from October 2022 to September 2024. A total of 269 adults (BMI ≥30 kg/m^2^ or ≥27 kg/m^2^ with obesity-related complications) who were prescribed Semaglutide or Tirzepatide as part of a comprehensive, multidisciplinary weight management program were included. Body composition was assessed using the InBody 370S analyzer. Data were extracted from the health information system. A repeated measures ANOVA was used to determine significance (p < 0.05) in the statistical analyses, which were conducted using SPSS v29.

**Results:**

Both medications significantly reduced weight, BMI, waist circumference, waist-to-height ratio, fat mass, and visceral fat (p < 0.001). At 6 months, weight loss was similar (−9.09 % vs −10.7 %), but by 12 months, Tirzepatide achieved greater reduction (22.02 % vs 11.59 %). Both improved glycemic control and liver function. Females exhibited greater weight loss. Lifestyle interventions supported skeletal muscle mass preservation.

**Conclusion:**

Semaglutide and Tirzepatide, significantly improved body composition, weight loss, and metabolic parameters in people with obesity. Tirzepatide demonstrated greater long-term efficacy.

## Graphical abstract

**Figure:**
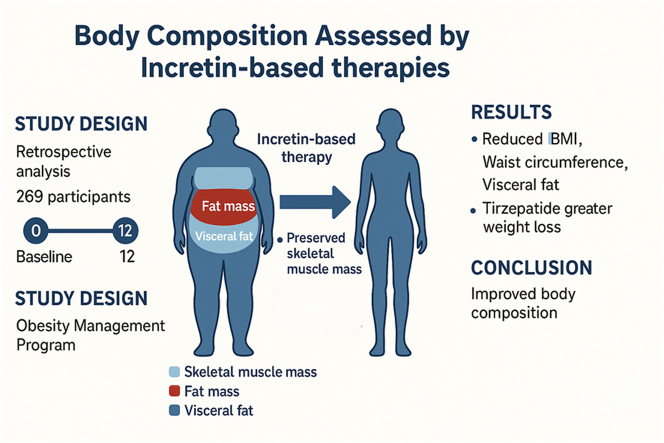

## 1 Introduction

The global epidemics of overweight and obesity pose serious health challenges for individuals and the healthcare systems worldwide [1]. The prevalence of obesity in adults has significantly increased in the Arabian Gulf, with estimates for women and men ranging from 17 % to 48 % and 8 %–36 % respectively [2]. A recent cross-sectional, multi-center, population-based study reported an overall prevalence of obesity at 28 % and overweight at 35.4 % indicating that 63.4 % of the population were living with either overweight or obesity. The burden was significantly higher among UAE nationals (35.5 %) compared to non-UAE nationals, notably 18.8 % from Southeast Asian region and 25.9 % from rest of the world. The prevalence of obesity was higher in women (30.4 %) than in men (25.9 %) [3].

A body mass index (BMI) of 25–30 kg/m2 is considered overweight, whereas BMI of ≥ 30 kg/m2, is considered as having obesity [4]. Increased body weight and BMI are associated with increased risk of cardiovascular disease and other metabolic conditions [5]. In addition to BMI, waist circumference, and visceral fat are associated with increased cardiometabolic risk [6].

Current endocrine guidelines recommend the use of pharmacotherapy for obesity management in conjunction with medical nutrition therapy, physical activity, and/or psychological interventions [7,8]. In people living with overweight/obesity, a sustained weight loss of more than 10 % can have significant health benefits, including management and prevention of conditions like type 2 diabetes, hypertension, metabolic dysfunction associated fatty liver disease, and obstructive sleep apnea, in addition to improving overall quality of life [9].

The following incretin-based medications are approved for weight management in people with obesity (BMI ≥30) or those who are overweight (BMI ≥27) with at least one weight-related condition, such as type 2 diabetes or hypertension. Liraglutide is a once daily glucagon‐like peptide‐1 receptor agonist (GLP-1RA) that received approval from the food and drug administration (FDA) in December 2014. This was followed by Semaglutide, a once weekly (GLP-1 receptor agonists) which was approved in June 2021 [10]. Tirzepatide is a dual once weekly glucagon‐like peptide‐1 receptor agonist (GLP-1RA) and glucose‐dependent insulinotropic polypeptide (GIP) approved by FDA in November 2023 [11]. All of these incretin based obesity medications are injectable, subcutaneously administered agents.

Randomized controlled trials (RCTs) offer insights into a drug's efficacy under ideal conditions, whereas real-world evidence (RWE) studies demonstrate a drug's effectiveness in typical clinical practice among a diverse patient population across various clinical scenarios [12]. Therefore, RWE studies can complement the findings of RCTs and help provide a complete picture of the advantages and disadvantages of medications during real-world use.

Despite the growing global and regional burden of obesity, there has been limited evaluation of the real-world effectiveness of these new pharmacological treatments such as Semaglutide and Tirzepatide in the Middle East. The percentage change in body weight from baseline to 12 months was the main primary outcome of this study. Thus, the purpose of this study was to examine the effects of Semaglutide and Tirzepatide on body composition in people with overweight and obesity who were enrolled in a multidisciplinary weight management program for a year.

## 2 Methods

### 2.1 Study design

This retrospective observational study evaluated once weekly incretin-based obesity medications in adult patients with overweight and obesity who were receiving routine clinical care at Genesis Healthcare Centre in Dubai, UAE from October 2022 to September 2024.

### 2.2 Definitions

Body mass index (BMI) was defined as weight divided by height squared (kg/m2). Waist circumference (WC), was defined as measurement midway between the lowest rib and the iliac crest using a flexible tape. Waist-to-height ratios (WHR) were obtained by dividing waist circumference by height. Body composition was assessed using the InBody 370S body composition analyzer (InBody Co., Ltd., USA), a multi-frequency bioelectrical impedance analysis (BIA) device. It uses tetrapolar 8-point tactile electrode system with hand and foot electrodes; there are no abdominal contact electrodes on this model. Participants were measured barefoot on the foot electrodes while gripping the hand electrodes with arms slightly abducted, as per manufacturer instructions. The analyzer estimates body composition parameters, such as total body fat mass (kg), skeletal muscle mass (kg), percent body fat (%), and visceral fat level using direct segmental multi-frequency BIA (5, 50, and 250 kHz).

### 2.3 Study population

Adults ≥ 18 years who were enrolled in the European Association for the Study of Obesity (EASO)-accredited Collaborating Centers for Obesity Management (COMs) multidisciplinary weight management program were eligible for inclusion. If a patient had a body mass index (BMI) ≥30 kg/m^2^, or ≥27 kg/m^2^ with at least one obesity-related complication (such as type 2 diabetes, hypertension, dyslipidemia, obstructive sleep apnea, or osteoarthritis), they were eligible for use of obesity medications (OMs). Patients were excluded from the study if they met any of the following criteria: pregnancy, lactation or inability to comply with follow up assessments. Patients with longstanding or complicated type 2 diabetes already under endocrine care were not routinely referred to the program and were not included. Patients received standard care including lifestyle intervention advice (increased protein intake and strength training twice a week) and follow up in clinic for weight management at 3, 6, 9 and 12 months. The standard dose escalation schedule was used for both obesity medications. The obesity medications were continued until the end of the follow up period.

### 2.4 Data collection

Data was collected from health information system Credence® (Sapiens Software Systems LLC, Dubai, UAE) and the In Body 370 S body composition analyzer (InBody Co., Ltd., USA).

The primary endpoint was the change from baseline to 12 months in body weight percentage. The secondary endpoints included: the change from baseline to 12 months in waist circumference (WC) (cm), waist height ratio (WHR), body fat mass (kg and %), skeletal muscle mass (kg) and visceral fat level. The percentage change in body weight was calculated as (follow-up weight − baseline weight)/baseline weight. The clinical parameters included glycated haemoglobin (HbA1c), renal function such as creatinine and estimated glomerular filtration rate (eGFR), liver function tests including aspartate aminotransferase (AST), alanine aminotransferase (ALT), gamma glutamyl transpeptidase (GGT) and lipid profile including total cholesterol, triglycerides, low-density lipoprotein (LDL) and high-density lipoprotein (HDL).

Discontinuation of Medications: For primary prevention, people with obesity showing sustained weight loss, clinician considered discontinuation of statins when lipid profile showed LDL-C, Triglycrides and HDL-C within guideline concordant low-risk ranges and it remained controlled for at least 3 months following discontinuation of statins. Antihypertensives and metformin were similarly reviewed using standard thresholds (BP ≤ 130/80 mmHg and HbA1c ≤ 5.7 %)

### 2.5 Statistical methods

Key demographics, anthropometrics, and clinical data were analyzed using IBM Statistical Package for Social Sciences software version 29 (SPSS Inc., Chicago, IL). Categorical variables were cross tabulated to examine the independence between variables; for such variables, the chi-square test was used. Kolmogorov-Smirnov was used to evaluate the normality of continuous variables. The Kruskal-Wallis's test (H test) was used to determine if there are statistically significant differences between two or more groups of an independent variable on a continuous or dependent. The data were displayed as n (%) or mean ± standard deviation and the repeated measure ANOVA p-value was given. All statistical analyses were deemed significant if the P-value was less than 0.05.

### 2.6 Ethical statement

Ethical approvals were obtained from the local Research and ethics committee, King's College Hospital Dubai, UAE (KCH/MOI/740).

## 3 Results

The study included 269 patients in total. The majority (78.1 %) were female, and the mean age was 41.2 ± 9.04 years. The patients were from 45 different nationalities, representing a heterogeneous population. *The most prevalent obesity related complication was dyslipidemia (65.4 %), prediabetes (23 %) followed by metabolic dysfunction associated fatty liver disease (20 %).* The mean (SD) BMI was 32.64 ± 5.47 kg/m^2^ and mean weight was 91.28 ± 19.22 kg at baseline. Regarding pharmacologic therapy, Tirzepatide was prescribed to 125 patients, Semaglutide to 121 patients and Liraglutide to 8 patients. A summary of baseline demographics and clinical characteristics are shown in Table 1.

**Table 1:** Baseline Demographics and clinical characteristics of patients.

Variables	Total (N = 269) Mean ± SD or n (%)	Semaglutide (N = 121)	Tirzepatide (N = 125)	p-value
**Age (years)**	41.20 ± 9.04	40.56 ± 9.73	41.81 ± 8.32	0.28
**Male**	54 (21.9 %)	18 (33.3 %)	36 (66.7 %)	0.008
**Female**	192 (78.1 %)	103(53.6 %)	89(46.4 %)	0.008
**Ethnicity**				
**Asian**	74 (27.5 %)	38 (51.3 %)	32 (43.2 %)	0.184
**American & Canadian**	13 (4.8 %)	8 (61.5 %)	5 (38.5 %)	
**Arabs**	28 (10 %)	9 (32.1 %)	17(60.7 %)	
**Africa**	37 (13.8 %)	10 (27 %)	21(56.7 %)	
**European**	99 (36.8 %)	52 (52.5 %)	40 (40.4 %)	
**New Zealand & Australia**	14 (5.2 %)	4 (28.5 %)	7 (50 %)	
**Missing**	5 (1.8 %)	2	1	
**Obesity related complications**				
**Prediabetes**	53 (23 %)	22 (41.5 %)	28 (52.8 %)	0.052
**Type 2 Diabetes**	5 (1.8 %)	0	5 (1.8 %)	
**Hypertension**	20 (8.1 %)	3 (15 %)	17 (85 %)	**0.001**
**Dyslipidemia**	176 (65.4 %)	78 (44.3 %)	84 (47.7 %)	0.982
**MAFLD**	54 (20 %)	13 (24.1 %)	35 (64.8 %)	**<0.001**
**Obstructive Sleep Apnea (OSA)**	14 (5.2 %)	5 (35.7 %)	7 (50 %)	0.593
**Polycystic ovarian syndrome (PCOS)**	34 (12.6 %)	22 (64.7 %)	10 (29.4 %)	0.039
**Weight (kg)**	91.28 ± 19.22	86.72 ± 13.91	97.52 ± 18.54	**<0.001**
**BMI (kg/m^2^)**	32.64 ± 5.47	30.52 ± 3.43	34.03 ± 5.25	**<0.001**

**Note:** Data were presented as n (%) or mean ± standard deviation. The p-value from ANOVA or chi-squared test where appropriate. P -values <0.001 were considered as statistically significant, the bold text highlights the significant differences between studied groups (P values 0.001).

**Abbreviations:** PCOS, Polycystic ovarian syndrome; OSA, Obstructive Sleep Apnea.

Table 2 shows changes in anthropometric parameters at baseline, 6 and 12 months stratified by the obesity medications prescribed (Semaglutide and Tirzepatide). Both medications were associated with statistically significant reduction in all anthropometric measures including body weight, BMI, waist circumference, waist to height ratio, fat mass and visceral fat (p < 0.001).

**Table 2:** Anthropometrics measurements at baseline, 6 and 12 months.

Variable	Drug	Baseline N = 269 Mean ± SD	6 Months N = 198 Mean ± SD	12 Months N = 94 Mean ± SD	p-value
**Weight (kg)**	Semaglutide	86.72 ± 13.91	78.39 ± 14.06	75.37 ± 15.54	**<0.001**
	Tirzepatide	97.52 ± 18.54	87.40 ± 17.00	82.36 ± 17.12	
**BMI (kg/m^2^)**	Semaglutide	30.52 ± 3.43	27.55 ± 3.54	26.46 ± 4.07	**<0.001**
	Tirzepatide	34.03 ± 5.25	30.55 ± 4.81	28.74 ± 5.03	
**Waist Circumference (cm)**	Semaglutide	97.03 ± 9.02	88.16 ± 8.53	84.63 ± 8.88	**<0.001**
	Tirzepatide	102.79 ± 11.72	92.14 ± 10.54	88.56 ± 11.36	
**WHR**	Semaglutide	0.582 ± 0.04	0.528 ± 0.04	0.507 ± 0.04	**<0.001**
	Tirzepatide	0.614 ± 0.06	0.551 ± 0.05	0.529 ± 0.06	
**Fat Mass (kg)**	Semaglutide	35.30 ± 8.56	28.89 ± 8.80	26.23 ± 9.29	**<0.001**
	Tirzepatide	42.46 ± 11.77	34.84 ± 11.72	30.48 ± 11.32	
**%Fat**	Semaglutide	40.68 ± 6.48	35.58 ± 9.66	34.45 ± 7.29	0.134
	Tirzepatide	42.78 ± 7.00	39.33 ± 7.88	36.55 ± 8.33	
**SMM (kg)**	Semaglutide	28.34 ± 5.97	27.06 ± 6.07	26.90 ± 6.21	**<0.001**
	Tirzepatide	31.72 ± 9.45	29.28 ± 6.30	29.28 ± 8.86	
**Visceral Fat**	Semaglutide	16.22 ± 3.14	13.22 ± 4.05	11.61 ± 4.21	**<0.001**
	Tirzepatide	17.80 ± 2.87	15.20 ± 4.26	13.39 ± 4.49	

**Note:** Data were presented as n (%) or mean ± standard deviation. The p-value from ANOVA or chi-squared test where appropriate.P -values <0.001 were considered as statistically significant, the bold text highlights the significant differences between studied groups (P values 0.001).

**Abbreviations:** BMI, body mass index; WHR, waist-to-hip ratio; SMM, skeletal mass muscle.

Laboratory parameters were also compared between people with obesity based on the use of different obesity medications. AST and ALT levels improved significantly, with normalization observed at both 6 and 12 months following treatment with either medication. (Table 3).

**Table 3:** Laboratory investigations at baseline, 6 and 12 months.

Variable	Drug	Baseline Mean ± SD	6 Months Mean ± SD	12 Months Mean ± SD	p-value
**HbA1c (%)**	Semaglutide	5.25 ± 0.43	4.88 ± 0.28	5.02 ± 0.28	**<0.001**
	Tirzepatide	5.50 ± 0.47	4.99 ± 0.37	4.90 ± 0.39	
**ALT (U/L)**	Semaglutide	27.78 ± 14.41	20.89 ± 9.32	22.95 ± 12.07	0.003
	Tirzepatide	26.63 ± 15.01	23.34 ± 14.45	20.80 ± 10.62	
**AST (U/L)**	Semaglutide	34.75 ± 4.25	21.97 ± 5.71	22.66 ± 6.28	**<0.001**
	Tirzepatide	22.47 ± 5.96	23.17 ± 7.78	21.29 ± 5.90	
**GGT (U/L)**	Semaglutide	23.85 ± 19.19	23.70 ± 27.96	23.51 ± 22.13	0.901
	Tirzepatide	30.18 ± 26.43	25.22 ± 23.24	19.92 ± 16.35	
**Total Cholesterol (mg/dL)**	Semaglutide	222.92 ± 48.64	194.36 ± 61.20	194.60 ± 30.97	0.137
	Tirzepatide	217.42 ± 41.20	194.73 ± 33.65	190.73 ± 29.87	
**LDL (mg/dL)**	Semaglutide	140.88 ± 36.55	124.12 ± 44.82	115.95 ± 31.58	0.131
	Tirzepatide	137.72 ± 39.76	121.53 ± 33.26	114.36 ± 31.40	
**HDL (mg/dL)**	Semaglutide	60.94 ± 16.49	55.54 ± 18.27	60.61 ± 12.58	0.131
	Tirzepatide	50.83 ± 10.46	49.40 ± 9.57	52.63 ± 11.85	
**Triglycerides**	Semaglutide	112.32 ± 60.54	92.52 ± 38.47	102.92 ± 51.66	0.148
	Tirzepatide	149.39 ± 66.78	121.43 ± 46.74	115.69 ± 58.86	
**Creatinine**	Semaglutide	64.40 ± 14.02	67.53 ± 12.34	62.58 ± 12.18	0.432
	Tirzepatide	65.20 ± 10.28	68.48 ± 12.81	67.48 ± 10.34	
**eGFR**	Semaglutide	105.37 ± 10.27	100.75 ± 11.80	108.13 ± 6.78	0.769
	Tirzepatide	108.06 ± 10.82	105.00 ± 14.64	106.23 ± 12.44	

**Note:** Data were presented as n (%) or mean ± standard deviation and the p-value expressed from repeated measure ANOVA. The P -values <0.001 were considered as statistically significant, the bold text highlights the significant differences between studied groups (P values < 0.001).

**Abbreviations:** ALT, Alanine aminotransferase; AST, Aspartate aminotransferase; GGT, gamma-glutamyl transferase; LDL, low-density lipoprotein; HDL, high-density lipoproteins; eGFR, glomerular filtration rate.

At baseline, three patients with prediabetes and one patient with type 2 diabetes were receiving metformin, all of whom were able to discontinue therapy at 12 months. Among eighteen patients on one to two antihypertensive medications (ACE inhibitors/calcium channel blockers/beta blockers) and only three patients required ongoing monotherapy and two patients required dual therapy at 12 months. Of 14 patients on statins, eight were able to discontinue lipid-lowering statins at 12 months. Additionally, 10 patients were using continuous positive airway pressure (CPAP) for obstructive sleep apnea, of whom only one patient continued CPAP treatment at 12 months follow -up.

**Fig. 1:**
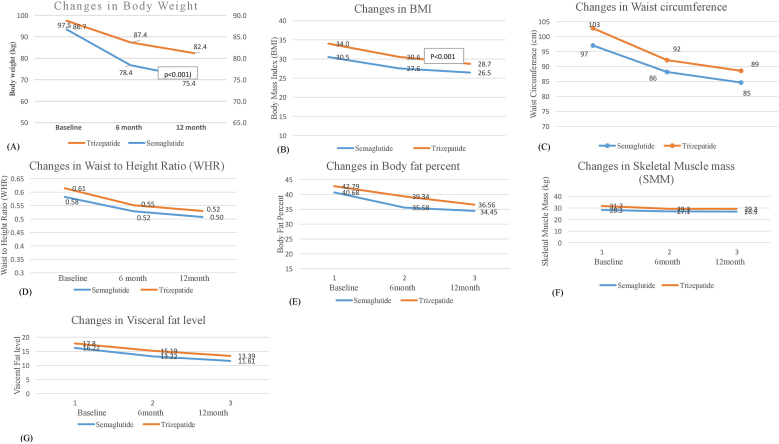
Change in Anthropometrics from baseline to 6 and 12 months with Semaglutide and Tirzepatide. (A) Changes in Body Weight (B) Changes in BMI. (C) Changes in Waist Circumference. (D) Changes in Waist to Height Ratio. (E) Changes in Body fat percent. (F) Changes in Skeletal Muscle Mass. (G) Changes in Visceral fat level.

**Fig. 2:**
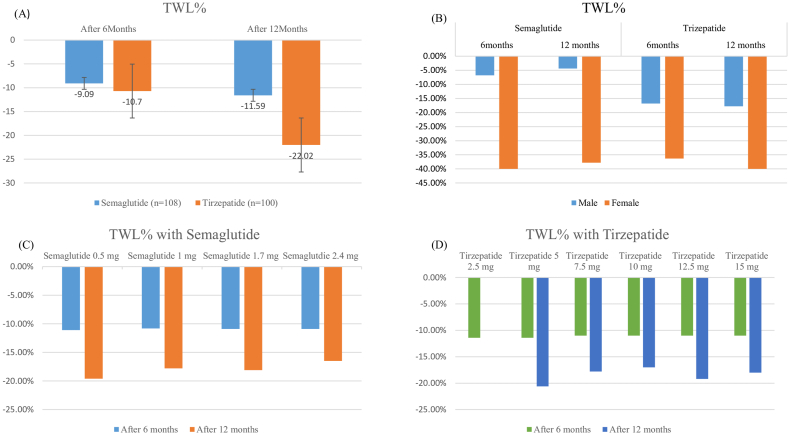
(A) Total weight loss (TWL) in % 6 and 12 months from baseline with Semaglutide and Tirzepatide. (B) Total weight loss (TWL) in % 6 and 12 months from baseline with Semaglutide and Tirzepatide based on gender. (C) Total weight loss (TWL) in % 6 and 12 months from baseline with Semaglutide based on dosage. (D) Total weight loss (TWL) in % 6 and 12 months from baseline with Tirzepatide based on dosage.

Fig. 1 illustrates changes in anthropometric parameters (weight, BMI, WC, WHR, Fat mass %, SMM and VF) at 6 and 12 months, comparing outcomes between Semaglutide and Tirzepatide. As shown in Fig. 2 a, the mean on-treatment change in body weight was −7.58 kg (−9.09 %) for Semaglutide vs −10.2 kg (−10.7 %) for Tirzepatide at 6 months. At 12 months, weight reductions increased to −9.64 kg (−11.59 %) with Semaglutide and −21.28 kg (−22.02 %) with Tirzepatide, indicating a significantly greater long-term effect with Tirzepatide.

## 4 Discussion

This real-world observational study enrolled 269 patients in a multidisciplinary weight management program to evaluate the use of different incretin-based obesity medications in people with obesity. Majority were females (78.1 %) reflecting higher prevalence of obesity in females as reported recently in UAE [3]. They represented more than 45 nationalities reflecting diversity in UAE which has more than 200 nationalities. The majority of participants were European (36.8 %) followed by Asians (27.5 %), and the mean age was 41.2 ± 9.04 years. This finding is in line with a recent population-based study that showed that, as a result of decreased physical activity and lifestyle modifications, the overall prevalence of obesity was higher among adults aged 40–59 years from Southeast Asian region (33.8 %) and among adults aged >60 years from other countries (36.2 %) [3].

Over a 12-month period, our study showed statistically significant decrease in waist circumference, WHR, body fat% and visceral fat. There was an initial decline in SMM (kg) during the first 6 months but then it plateaued in the subsequent 6 months. This stabilization is attributable to nutritional and physical activity interventions advised as part of the program-specifically as patients were advised to increase the protein intake to 60–90 g/day and incorporating strength training at least twice a week. These strategies are consistent with methods known to preserve lean body mass during weight loss. A mean weight decrease of 9.55 kg was reported in a 24-week retrospective study involving 88 patients with type 2 diabetes and obesity. This was mostly due to fat mass reduction using bioelectrical impedance analysis with subcutaneous Semaglutide [13]. Similarly, Tirzepatide reduced fat mass far more than lean mass from baseline compared to placebo (estimated treatment difference of −25.7 % for fat mass vs −8.3 % for lean mass at week 72), according to a subgroup analysis from 160 patients in SURMOUNT-1 trial. The proportion of weight reduction was 74 % as fat mass loss and 26 % as lean mass loss, similar to that with placebo (75 % and 25 %, respectively) [14].

In this study, participants receiving Semaglutide experience a mean body weight reduction of 9.09 % at 6 months and 11.59 % at 12 months (52 weeks). These results align with a real-world retrospective study that demonstrated a 10 % weight loss with Semaglutide in six months [15]. In the STEP 1 study, the group treated with Semaglutide showed a 14.9 % reduction in body weight from baseline to week 68 [16].

Tirzepatide-treated participants in our cohort achieved a 10.7 % reduction in body weight in 6 months, increasing to 22.02 % in 12 months, suggesting a more pronounced long-term benefit. This is similar to findings from a recent retrospective study on 239 patients which reported 11.6 % reduction in body weight in 6 months with Tirzepatide treatment [17]. With 10 mg and 15 mg dosages of Tirzepatide in SURMOUNT 1 trial, in people with obesity, the average weight reductions were19.5 % and 20.9 % respectively at 72 weeks [18].

With both Semaglutide and Tirzepatide, females showed significantly greater weight loss than males in our subgroup analyses. This trend is consistent with gender-based subgroup analysis from SURMOUNT1-3 trials, which also reported significantly greater weight reductions in females compared to males, across all doses of Tirzepatide. In these trials, Tirzepatide treatment led to weight loss ranging from −11.5 % to −27.6 % in females, compared to −8.8 % to −18.9 % in males. This was presented at 60th The European Association for the Study of Diabetes (EASD), Madrid (9–13 Sept) [19]. These results reinforce the observation that biological sex may influence responsiveness to incretin-based therapies, potentially due to differences in body composition, hormonal regulation, or metabolic rate.

In our subgroup analysis, TWL % was 11 % across all patients at 6 months regardless of whether patients were on Semaglutide (any dose from 0.5 mg to 2.4 mg) or Tirzepatide (5–15 mg). This indicates that both GLP-1 and dual GIP/GLP-1 agonists resulted in similar weight loss outcomes by a 6-month mark. However, in 12 months, participants on Semaglutide 0.5 mg experienced a 19.6 % reduction in body weight, while those on 2.4 mg had a lower reduction of 16.5 %. The observed greater effectiveness of 0.5 mg semaglutide likely reflects real-world treatment dynamics rather than a true inverse pharmacologic dose-response. By comparison, Tirzepatide showed more consistent efficacy, resulting in weight reduction that ranged from 18 % to 20 % across doses from 5 to 15 mg. This shows that there is a variability between efficacy of medications at 12 months. This may indicate adherence at higher doses of Semaglutide leading to dose reduction or whether these patients had different severity of metabolic disease. However, since this was an observational study conducted in real world practice, factors such as treatment compliance and dosage modifications were not analyzed and should be examined further in prospective studies in the future.

This is the only real-world observational study to date, evaluating changes in body weight in patients who are overweight or obese and receiving treatment with both Semaglutide and Tirzepatide as part of a comprehensive weight management program. Our results are consistent with SURMOUNT-5 trial which included 751 people with obesity or overweight and associated comorbidities. When compared to Semaglutide, Tirzepatide resulted in mean body weight loss of 20.2 % compared to 13.7 % [20]. Tirzepatide exhibits better sustained weight reduction after 12 months, despite the fact that early weight loss is comparable between GLP-1 and dual GIP/GLP-1 agonists at 6 months.

## 5 Limitations

This study was retrospective in design from single center and did not include control group, limiting our ability to draw causal inferences regarding the effect of treatment on the measured outcomes. In our study, body composition was measured using a standing, multi-frequency Bioelectrical impedance analysis (BIA) analyzer (InBody 370S). It used 8-point tactile electrode system with hand and foot electrodes; there were no abdominal contact electrodes on this model. However, hand/foot bioelectrical impedance devices lack precision in assessing visceral adiposity, especially in females and in some race/ethnicity groups such as Black adults.

Therefore, waist circumference was measured separately using flexible tape measure.

Although BIA is frequently used in clinical practice because it is non-invasive and convenient, it is not considered the gold standard. The lack of precision in assessing visceral adiposity, especially in individual females, can somewhat be mitigated by (a) using BIA devices with abdominal touchpoints that better capture truncal impedance (validated against MRI) [21] (b) augmentation with ultrasound-derived abdominal measures; and (c) combining measured waist circumference with sex-specific visceral adipose tissue formulae [22].

Additionally, this analysis did not account for medication adherence, persistence, or adverse effects, which are important real-world factors that could significantly influence treatment outcomes.

## 6 Conclusions

The use of obesity medications was associated with significant improvements in body composition and anthropometrics including WC, WHR, fat% and VF (*P* < 0.01). By including twice weekly strength training and a sufficient protein intake, our cohort showed that skeletal muscle mass was maintained from 6 to 12 months. The mean total weight loss was 9.09 % with Semaglutide at 6 months and 10.7 % at 12 months compared to 11.59 % with Tirzepatide at 6 months and 22.02 % at 12 months. However, early weight loss was comparable between both medications at 6 months. Tirzepatide demonstrated superior weight reduction at 12 months. Additionally, females experienced greater weight loss than males, consistent with trends reported in clinical trials.

In addition to weight loss, the use of obesity medications was associated with significant improvement in glycaemic control and liver function tests(*P* < 0.01) suggesting wider metabolic benefits.

Significantly, a number of patients were able to discontinue medications used for obesity-related complications by the 12-month mark, highlighting the potential therapeutic benefits of pharmacologic obesity treatment in conjunction with lifestyle modification.

Take away Messages:

• Incretin-based obesity medications (OMs) produced clinically meaningful weight loss and improvements in anthropometrics like WC, WHR, body fat% and visceral fat in real-world weight management program.
• Preserving skeletal muscle mass is feasible when obesity medications are combined with twice weekly resistance training and adequate protein intake (approx. 60–90 gm/day), in our cohort skeletal muscle was maintained from 6 to 12 months.

## Author contributions

RB, AS, BMM, RK, SA conceptualized this work. All authors made a significant contribution to the work reported, whether that is in the conception, study design, execution, acquisition of data, analysis and interpretation, or in all these areas. They participated in drafting, revising or critically reviewing the article; approval the final version to be published; agreed on the journal to which the article has been submitted; and accepted accountable for all aspects of the work.

## Disclosures

RB, AS, BMM, RK, SA declare that they have no competing interests.

## Ethical statement

Ethical approvals were obtained from the local Research and ethics committee, King's College Hospital Dubai, UAE (KCH/MOI/740).

## Consent

Prior to their participation, all individual participants provided informed consent. Participants were informed that the information collected during the study as well as the study results may be published. We have assured them that their privacy and confidentiality will be protected, and no information that could be traced back to them will be disclosed.

## Data availability statement

Data were used under license at study sites and thus can-not be made publicly available. The source code used to perform data management and statistical analyses is made. The manuscript is aligned with the Reporting of Studies Conducted using Observational Routinely Collected Health Data for statement record.

## Declaration of use of Artificial Intelligence (AI)

The authors used a generative AI tool (ChatGPT) only for language editing. No AI tools were used to generate, analyse or interpret data, neither to create figures/tables from raw data; or to draw scientific conclusions.

## Funding information

None.

## Declaration of competing interest

None.

## Abbreviations

Body mass index (BMI), Waist circumference (WC), Waist height ratio (WHR), Skeletal muscle mass (SMM), Total body weight loss (TWL), Obesity management medications (OMMs).

## References

[1] World Health Organization Prevalence of obesity (Article online). https://www.who.int/diabetes/country-profiles/are_en.pdf.

[2] Mamdouh H., Hussain H.H.Y., Ibrahim G.M. (2023). Prevalence and associated risk factors of overweight and obesity among adult population in Dubai: a population-based cross-sectional survey in Dubai, the United Arab Emirates. BMJ Open.

[3] Abdelgadir E., Rashid F., Bashier A. (2025). Prevalence of overweight and obesity in adults from the Middle East: a large-scale population-based study. Diabetes Obes Metabol.

[4] Flegal K.M., Kit B.K., Orpana H. (2013). Association of all-cause mortality with overweight and obesity using standard body mass index categories: a systematic review and meta-analysis. JAMA.

[5] Georgoulis M., Damigou E., Chrysohoou C. (2024). Increased body weight and central adiposity markers are positively associated with the 20-year incidence of cardiovascular disease: the ATTICA epidemiological study (2002-2022). Nutr Res.

[6] Bhatti R., Warshow U., Joumaa M. (2021). Relevance of anthropometric measurements in a multiethnic obesity cohort: observational study. Interact J Med Res.

[7] Garvey W.T., Mechanick J.I., Brett E.M. (2016). American Association of Clinical Endocrinologists and American College of endocrinology comprehensive clinical practice guidelines for medical care of patients with obesity. Endocr Pract.

[8] Pedersen S.D., Manjoo P., Wharton S. (2025). Canadian adult obesity clinical practice guidelines: pharmacotherapy for obesity management. https://obesitycanada.ca/guidelines/pharmacotherapy.

[9] Perdomo C.M., Cohen R.V., Sumithran P. (2023). Contemporary medical, device, and surgical therapies for obesity in adults. Lancet.

[10] Elmaleh-Sachs A., Schwartz J.L., Bramante C.T. (2023). Obesity management in adults: a review. JAMA.

[11] Abbasi J. (2023). FDA green-lights Tirzepatide, marketed as Zepbound, for chronic weight management. JAMA.

[12] Blonde L., Khunti K., Harris S.B. (2018). Interpretation and impact of real-world clinical data for the practicing clinician. Adv Ther.

[13] Rodríguez Jiménez B., Rodríguez de Vera Gómez P., Belmonte Lomas S. (2024). Transforming body composition with Semaglutide in adults with obesity and type 2 diabetes mellitus. Front Endocrinol (Lausanne).

[14] Look M., Dunn J.P., Kushner R.F. (2025). Body composition changes during weight reduction with Tirzepatide in the SURMOUNT-1 study of adults with obesity or overweight. Diabetes Obes Metabol.

[15] Ruseva A., Michalak W., Zhao Z., Fabricatore A. (2024). Semaglutide 2.4 mg clinical outcomes in patients with obesity or overweight in a real-world setting: a 6-month retrospective study in the United States (SCOPE). Obes Sci Pract.

[16] Wilding J.P.H., Batterham R.L., Calanna S. (2021). Once weekly semaglutide in adults with overweight or obesity. N Engl J Med.

[17] Hankosky E.R., Chinthammit C., Meeks A. (2025). Real-world use and effectiveness of Tirzepatide among individuals without type 2 diabetes: results from the Optum Market Clarity database. Diabetes Obes Metabol.

[18] Jastreboff A.M., Aronne L.J., Ahmad N.N. (2022). Tirzepatide once weekly for the treatment of obesity. N Engl J Med.

[19] Garcia-Perez L.E. (2024). 60th EASD annual meeting.

[20] Aronne L.J., Horn D.B., le Roux C.W., Ho W., Falcon B.L., Gomez Valderas E., SURMOUNT5 Trial Investigators (2025). Tirzepatide as compared with semaglutide for the treatment of obesity. N Engl J Med.

[21] Browning L.M., Mugridge O., Chatfield M.D., Dixon A.K., Aitken S.W., Joubert I., Prentice A.M., Jebb S.A. (2010 Dec). Validity of a new abdominal bioelectrical impedance device to measure abdominal and visceral fat: comparison with MRI. Obesity (Silver Spring).

[22] Hoffmann J., Thiele J., Kwast S. (2024). A new approach to quantify visceral fat via bioelectrical impedance analysis and ultrasound compared to MRI. Int J Obes.

